# Structurally distorted perovskite La_0.8_Sr_0.2_Mn_0.5_Co_0.5_O_3-δ_ by graphene nanoplatelet and their composite for supercapacitors with enhanced stability

**DOI:** 10.1038/s41598-022-14324-5

**Published:** 2022-06-16

**Authors:** Bo-Min Kim, Hyo-Young Kim, Sung-Wan Hong, Won Ho Choi, Young-Wan Ju, Jeeyoung Shin

**Affiliations:** 1 Department of Mechanical Systems Engineering, Sookmyung Women’s University, Seoul, 04310 Korea; 2 Department of Chemical Engineering, College of Engineering, Wonkwang University, Iksan, 54538 Jeonbuk Korea; 3 Department of Electronics Engineering, Sogang University, Seoul, 04107 Korea; 4 Institute of Advanced Materials and Systems, Sookmyung Women’s University, Seoul, 04310 Korea; 5 Nanoscale Sciences and Technology Institute, Wonkwang University, Iksan, 54538 Jeonbuk Korea

**Keywords:** Chemistry, Materials science

## Abstract

Supercapacitors are promising energy storage devices with high charging/discharging speeds and power densities. To improve their poor stability, we fabricated electrodes by integrating perovskite materials (La_0.8_Sr_0.2_Mn_0.5_Co_0.5_O_3-δ_, LSMCO) possessing redox reaction ability with graphene nanoplatelets exhibiting good electronic properties. One of the resultant composites (L25G70) demonstrated high capacitance and excellent capacitance retention (95% after 5000 cycles). These results are superior to other electrodes (L50G45 and L75G20) containing a larger ratio of LSMCO, even L75G20 did not exhibit supercapacitor behavior after 3000 cycles. GN can induce structural distortion in LSMCO, thereby the high amount of adsorbed oxygen per lattice oxygen can explain the best electrochemical performance of L25G70, while structural collapse rationalized the failure of L75G20. The findings of this study demonstrated that the use of LSMCO can improve the cycling stability of supercapacitors.

## Introduction

Supercapacitors are promising energy storage systems indispensable for diverse energy-related applications, such as portable electronics, hybrid electric vehicles, and large industrial equipment^1^, owing to their high power densities, high charging/discharging speeds, and long lifespans^2^. Depending on their charge storage mechanisms, supercapacitors can be divided into electric double-layer capacitors (EDLCs) and pseudocapacitors^3^. EDLCs, which typically use carbon-based materials with a high surface area, store charges at the large interface between the carbon and electrolyte. This results in a higher power density but a lower volumetric energy density than those of conventional batteries^4–7^. Pseudocapacitors could overcome the limitations of EDLCs as they exhibit a higher energy density than EDLCs by storing more charges through a faradaic redox reaction^4^.

However, it is difficult to simultaneously achieve a high energy density as well as stability in the pseudocapacitor. Researchers have devoted significant efforts to address this issue by making composites with pseudocapacitive materials and carbon-based materials. Transition metal compounds have been proven to improve electrochemical performance^8,9^. In particular, carbon-based materials such as activated carbon (AC) and carbon nanotubes (CNT) have made great strides as they ensure high stability in alkaline and acidic conditions due to their robust surface properties^10,11^. Graphene also has beneficial properties, such as high charge mobility, ballistic transport, and zero effective mass, owing to its large surface area, high aspect ratio, and excellent electrical conductivity^12,13^. Recently, graphene nanoplatelets (GNs) composed of several graphene layers have been considered a more processable alternative to single-layered graphene with similar properties. GNs can be easily dispersed in various solvents or matrices, thereby increasing the number of active sites without decreasing the surface conductivity or dead volume^14–17^. The developments of these carbon-based materials have been employed to significantly improve the energy density of composites with high stability; however, to the best of our knowledge, few studies have systematically investigated the behavior of the pseudocapacitive materials in the composites.

ABO_3_-type perovskite structures are attractive pseudocapacitive materials because of their good structural stability suitable for electrochemical applications^18,19^. The modulation of the A and B sites can regulate the electronic properties of the material, inducing anion vacancy as a charge storage site for achieving pseudocapacitance while maintaining structural stability. For example, the modulation of cations of LaMnO_3_ and LaSrMnO_3_ triggered anion-based intercalation and oxygen insertion by the anion vacancy effect^20,21^. La_x_Sr_1-x_CoO_3-δ_ (0.3 ≤ x ≤ 1) demonstrated a high cycling stability, with the retention of 97% and 80% of the initial specific capacitance after 2000 cycles^22^. La_0.8_Sr_0.2_Mn_0.5_Co_0.5_O_3-δ_ perovskite (LSMCO) showed that mixed-valence transition metal cations (Mn^4+^/Mn^3+^/Mn^2+^) can provide high electrical conductivity and maintain a large number of oxygen vacancies, resulting in fast oxygen ion diffusion^23^. The binding angle and length between Mn and oxygen ions have strong effects on the electron double exchange, which explains the good electron conductivity of the material^24^. Although the capacity of perovskite materials was slightly lower, they demonstrated remarkably stable cycling performances compared with those of conductive polymers and metal oxides. Therefore, perovskites with structural stability are anticipated to aid in maintaining long-term cycling stability.

In this study, we demonstrate the enhanced stability of composites consisting of GNs and LSMCO. GNs can induce structural distortion in LSMCO, thereby producing abundant anion vacancies that serve as a robust charge storage site. Although various structural distortions in the lattice arrangement have been employed to enlarge exposed active sites^25–27^, we show a new approach to inducing structural distortion of perovskite by encapsulating with GNs. Consequently, the best cycling stability of the composite electrode could be achieved when the amount of perovskite material was maintained under a threshold value.

## Results

### Morphological and structural characterization

**Figure 1:**
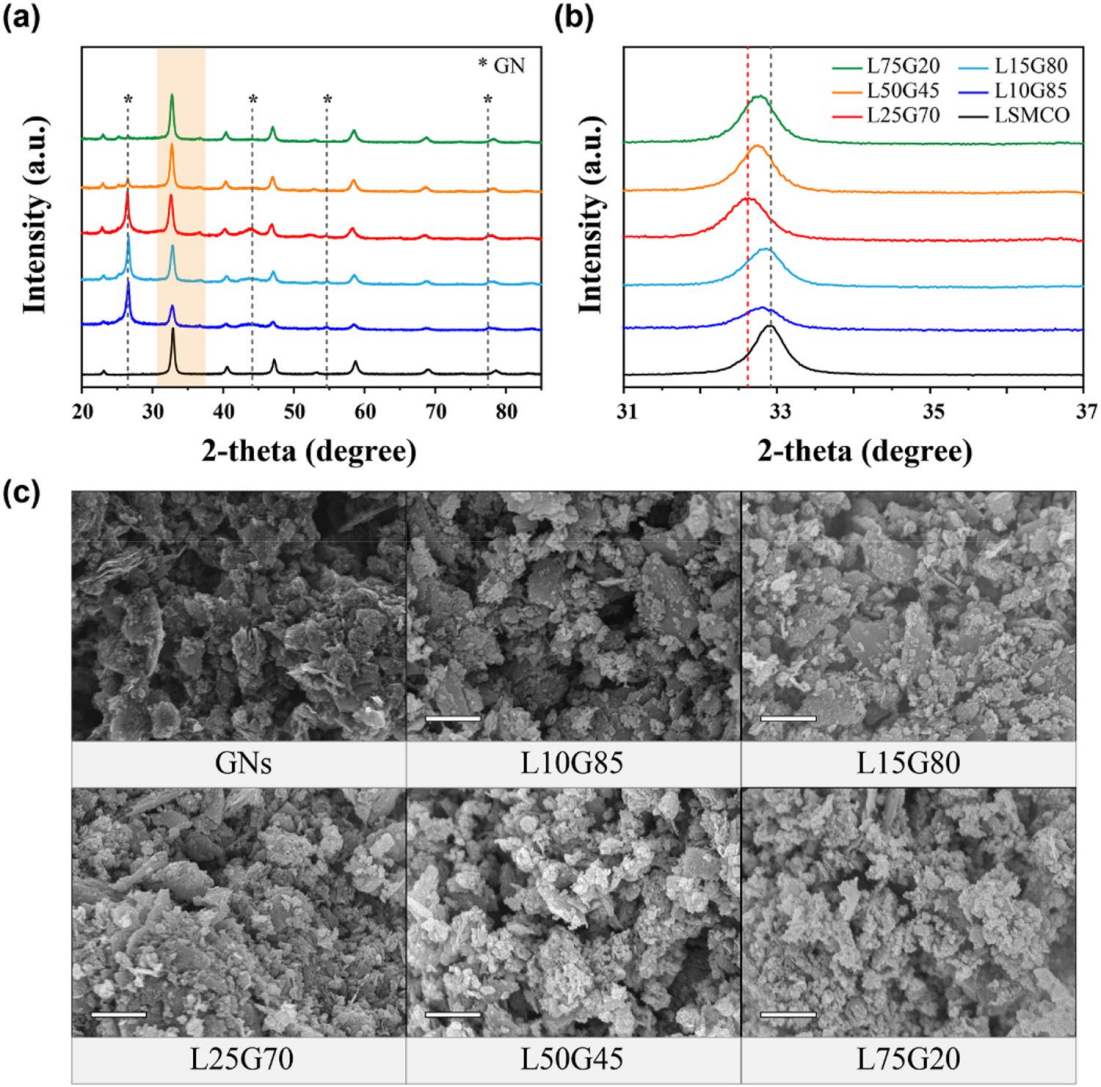
(**a**) X-ray diffraction (XRD) patterns, (**b**) partially enlarged XRD patterns, and (**c**) scanning electron microscopy (SEM) images of LSMCO, L10G85, L15G80, L25G70, L50G45, and L75G20. The XRD patterns of GN and LSMCO were from Ref. ^30^.

XRD patterns of LSMCO, L10G85, L15G80, L25G70, L50G45, and L75G20 are shown in Fig. 1a (when n and m are wt% of LSMCO and GNs in the LnGm composite, respectively). These patterns indicate that LSMCO in the composites had the same crystal structure as that in the pure state, despite the presence of GNs. Significantly, in the pattern of L25G70, the peak that was originally located at 32.90° in that of LSMCO shifted to the lowest angle (Fig. 1b). The d-spacing values of the corresponding peaks of L10G85, L15G80, L25G70, L50G45, and L75G20 are estimated to be 0.2729 (2θ = 32.79°), 0.2724 (32.85°), 0.2746 (32.58°), 0.2732 (32.75°), and 0.2729 nm (32.79°), respectively, all of which are larger than that of pristine LSMCO (0.2722 nm). This shows that the shift in the XRD peak is positively correlated to the in-plane strain of the LSMCO lattice^28,29^. In other words, an optimal amount of GNs in the composite with LSMCO can create a strong strain effect that results in the complete mixing of GNs and LSMCO without any separation.

SEM images in Fig. 1c clearly show that when the amount of LSMCO in the composite electrode was higher, more LSMCO particles existed in the GNs. A few LSMCO particles were observed in L10G85, L15G80, and L25G70. In L25G70, LSMCO was completely sandwiched between the GNs as indicated by the enhanced in-plane strain. Energy-dispersive X-ray spectroscopy (EDS) elemental mapping images of L25G70, L50G45, and L75G20 indicate the homogenous dispersion of LSMCO and GNs (Supplementary Fig. S1). When the amounts of GNs were not sufficient to completely enclose the LSMCO, as shown in the SEM images of L50G45 and L75G20, numerous small LSMCO particles were observed to have formed, suggesting induced distortion when a sufficient number of GNs was attached to the LSMCO. SEM and EDS images showed that LSMCO in L25G70 was still enclosed in GNs even after cycling tests unlike L50G45 and L75G20 (Supplementary Fig. S2). To demonstrate the distortion effect caused by added GNs, we compared the characteristics of three composites L25G70, L50G45, and L75G20.

**Figure 2:**
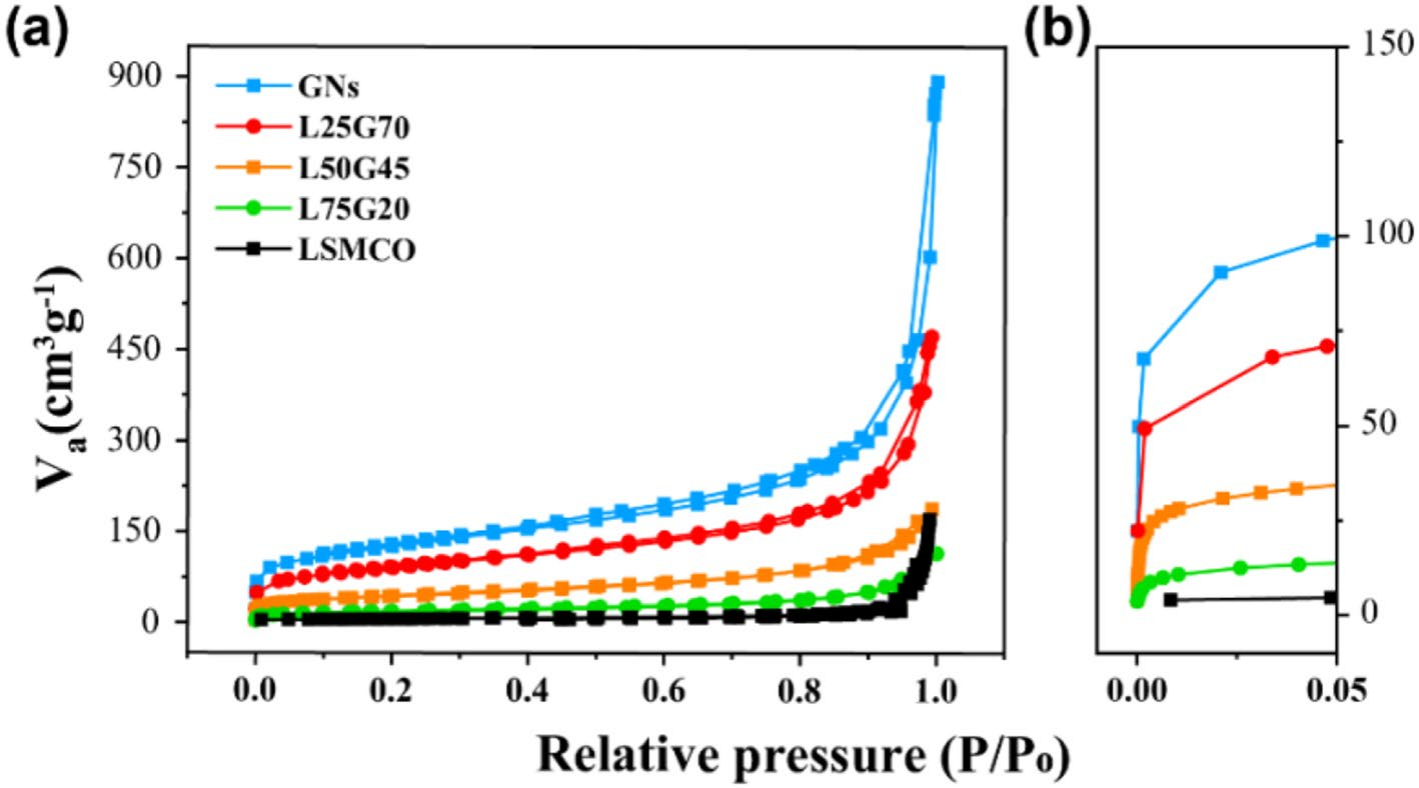
(**a**) Nitrogen adsorption–desorption isotherms (**b**) partially enlarged isotherms for GNs, L25G70, L50G45, L75G20, and LSMCO.

Brunauer-Emmett-Teller (BET) measurements were performed to determine the surface area, pore diameter, and pore volume of the composites, where V_a_ is the volume adsorbed. The N_2_ physisorption isotherms of L25G70 and L50G45 corresponded to a type II isotherm, but the isotherm of L75G20 exhibited a different shape (Fig. 2a). A type II isotherm indicates unrestricted mono-multilayer properties in the adsorption isotherm classification, and the interaction between adsorbed molecules is strong. In contrast, LSMCO exhibits a type III isotherm owing to weak adsorbate-adsorbent interactions when there is only negligible micro- and mesoporosity. The specific surface area of L25G70, L50G45, and L75G20 is 317.2, 156.6, and 66.8 m^2^ g^-1^, respectively. At relatively low pressure (< 0.1) as shown in Fig. 2b, adsorption is gradually reduced at a high amount of LSMCO, which is also reflected in the decreased surface area. Most of the surface area was estimated based on the amount of GNs (Table 1). While using 20 wt% GN, the pore volume decreased, indicating that a small amount of GNs penetrated the wide pores of LSMCO and blocked the space rather than encapsulating LSMCO.

**Table 1:** Specific surface area, average pore diameter, and total pore volume of all electrodes.

Sample	Specific surface area (m^2^ g^-1^)	Average pore diameter (nm)	Total pore volume (cm^3^ g^-1^)
GNs	441.8	8.6	0.67
L25G70	317.2	8.7	0.69
L50G45	156.6	7.2	0.28
L75G20	66.8	9.4	0.16
LSMCO	21.2	50.02	0.26

**Figure 3:**
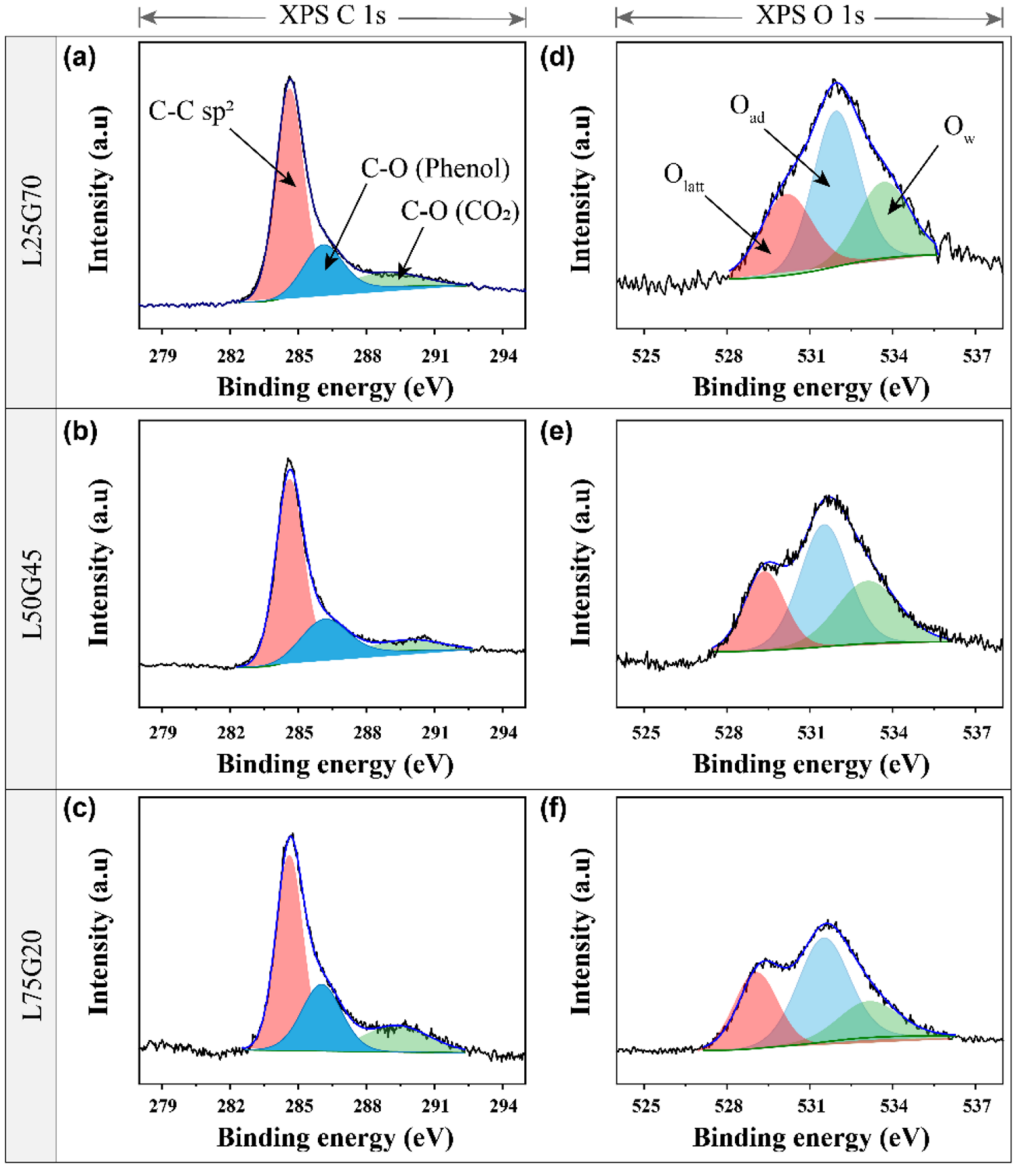
(**a**–**c**) Fitting results of X-ray photoelectron spectroscopy (XPS) C1s spectra and (**d**–**f**) O1s spectra of L25G70, L50G45, and L75G20.

XPS was used to analyze the chemical environment of the composites. C1s and O1s spectra for all electrodes are shown in Fig. 3. In these cases, the C1s spectrum can de deconvoluted into three peaks located at 284.6, 286.4, and ~ 290.1 eV, which are assigned to C–C sp^2^ bonds in the aromatic networks of graphene, C-O bonds in phenol groups, and carbonate or CO_2_, respectively. The O1s spectra showed peaks corresponding to lattice oxygen (O_latt_), adsorbed oxygen (O_ad_), and oxygen (O_w_) in water at binding energies of ~ 530.1, 531.9, and ~ 533.6 eV, respectively^31^. The ratios of O_ad_ to O_latt_ for different electrodes are 2.03 (L25G70), 1.86 (L50G45), and 1.55 (L75G20) while there is no noticeable difference between the carbon species. L25G70 has high oxygen adsorption depending on the oxygen lattice, indicating that LSMCO particles properly incorporated within GNs can utilize their enlarged interlayer space for oxygen adsorption.

### Electrochemical characterization

**Figure 4:**
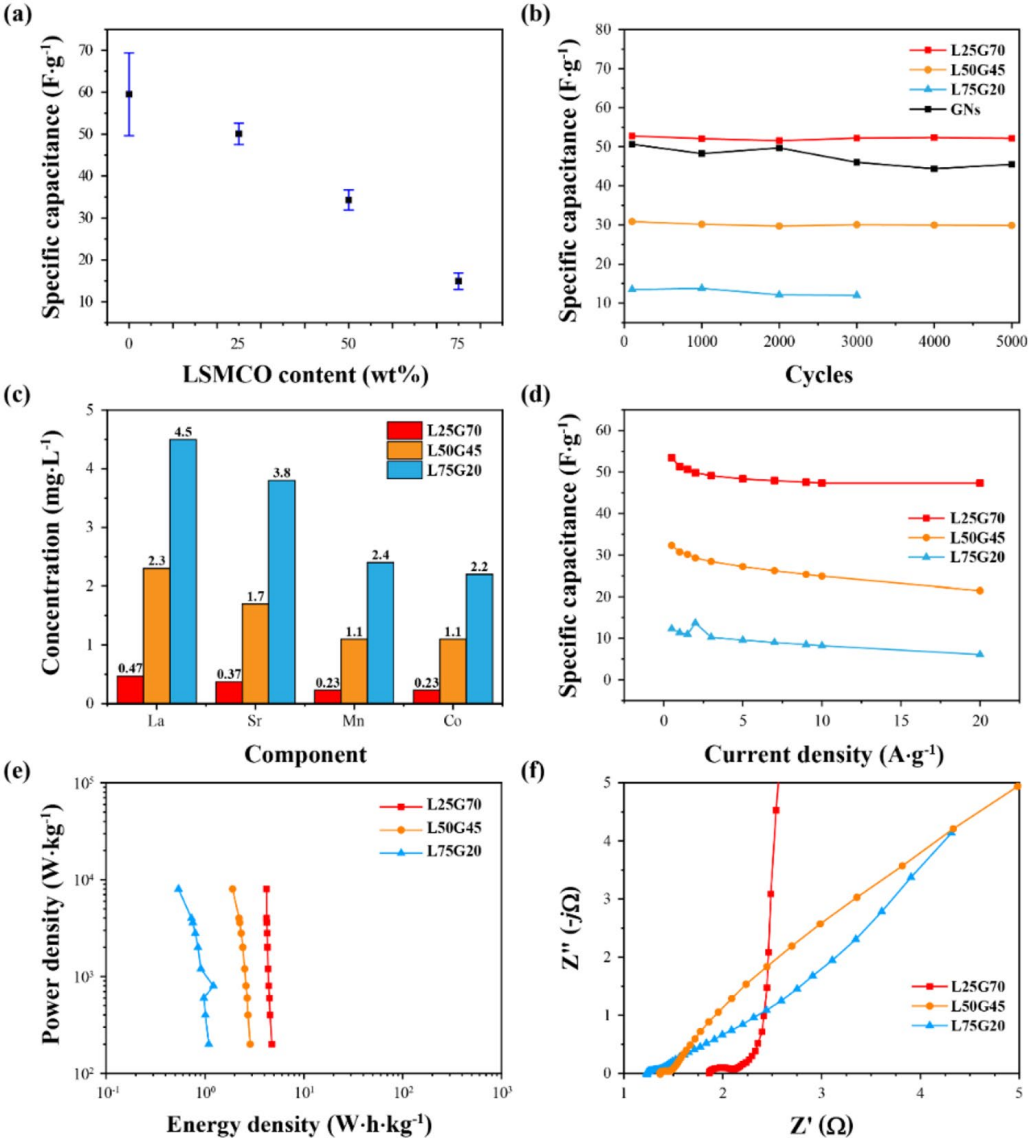
(**a**) Initial specific capacitance according to the LSMCO content (wt%) at the scan rate of 5 mV s^−1^. Markers and error bars represent the average and standard deviation, respectively. (**b**) The specific capacitance of L25G70, L50G45, L75G20, and GNs after the cycling test at 20 mV s^-1^, confirming the electrochemical stability of the LSMCO-based electrodes. (**c**) Metal concentrations dissolved in 1 M H_2_SO_4_ electrolyte from LSMCO-based electrodes, as measured using inductively coupled plasma-optical emission spectrometry (ICP-OES). (**d**) Variation of the specific capacitance with the current density for all electrodes. (**e**) Ragone plots. (**f**) Nyquist plots of all electrodes.

The electrochemical performances of three composites (L25G70, L50G45, and L75G20) and GNs were studied in a three-electrode cell using a 1.0 M H_2_SO_4_ electrolyte. CV was performed at a scan rate of 5 mV s^-1^ (Supplementary Fig. S3), and the specific capacitance as a function of the LSMCO content is shown in Fig. 4a. The average specific capacitance of GNs, L25G70, L50G45, and L75G20 was 59.55, 50.11, 34.28, and 14.90 F g^-1^ with the standard deviation of 9.91, 2.60, 2.41, and 1.95, respectively. The standard deviation of GNs was more than twice that of the others, while those of the composites remained small as the LSMCO content increased. The results indicate that the SO_4_^2-^ anion adsorption ability is not stable in the electrode using only GNs, despite sometimes higher capacitance than other composites. Figure 4b shows the changes in the specific capacitance during cycling. GNs exhibited unstable capacitive behavior, with a large variation range and nonlinear reduction compared to the composite electrodes. L25G70 and L50G45 maintained the specific capacitance as well as a small range of variation for each experiment. In contrast, L75G20 exhibited no supercapacitive behavior after 3000 cycles. These results suggest that an excessive amount of perovskite LSMCO undermines its stabilizing role through a possible interaction with the H_2_SO_4_ electrolyte. ICP-OES was used to study the dissolution of LSMCO components in the electrolyte. Figure 4c shows the contents of metals from LSMCO in the 1 M H_2_SO_4_ electrolyte after 5000 cycles (Supplementary Figs. S4, S5, S6 and S7). Because La in the perovskite material is readily dissolvable in H_2_SO_4_^32^, the concentration of La, Sr, Mn, and Co in the electrolyte increased when the electrode contained more LSMCO. As indicated by the amounts dissolved more than simply estimated amounts based on the wt% of the composites, the dissolution of a large amount of La in L75G20 can cause the structure to collapse, which explains the sudden failure of the L75G20 electrode during the cycling test.

The galvanostatic charge–discharge (GCD) curves of GN, L25G70, L50G45, and L75G20 are shown in Supplementary Figs. S8, S9, S10 and S11. The specific capacitance according to the current density was calculated using Eq. (2), and the results are shown in Fig. 4d. The specific capacitance of each electrode matched the corresponding CV measurements. At high current densities, the specific capacitance decreased for all electrodes owing to the limited ion diffusion rate and low ion penetration on the electrode surface^33,34^. These are the typical behaviors of supercapacitors. The pseudocapacitive materials are listed in Supplementary Fig. S12 to compare electrochemical performance. The Ragone plots of all electrodes are displayed in Fig. 4e. The gravimetric energy and power densities of all electrodes were calculated using Eqs. (3) and (4), respectively. The energy density of L25G70, L50G45, and L75G20 were in the range of 4.74–4.20, 2.87–1.91, and 1.09–0.54 Wh kg^-1^ when the current density varied between 0.5 and 20 A g^-1^, with a relative change of 11.42%, 33.60%, and 50.54%, respectively. Among them, L25G70 exhibited the largest lattice strain owing to the successful incorporation of LSMCO and GNs, which led to a high ratio of adsorbed oxygen as well as an abundant amount of SO_4_^2-^ adsorbed on the surface^35^.

Electrochemical impedance spectroscopy (EIS) is a useful tool for measuring the electrochemical behavior of electrodes, and typical Nyquist diagrams of each electrode are shown in Fig. 4f (Supplementary Fig. S13). The Nyquist plot consists of three regions. The high-frequency region describes series resistance (R_s_) caused by the resistances of the electrode and electrolyte. The diameter of the semicircular shape in the middle-frequency region is assumed to be equal to the charge transfer resistance (R_ct_). A Warburg impedance (slope of ~ 45°) at the low frequency is assigned to ion transport limitation in the electrolyte as equivalent distribution resistance; the straight vertical line at the low-frequency region is attributed to the dominant capacitive behavior of the EDLC at the electrode/electrolyte interface^36^. L50G45 and L75G20 with larger amounts of LSMCO showed smaller R_ct_ values, but the diffuse layer resistance was lower than those of the other two electrodes.

## Discussion

In summary, electrodes composed of different ratios of perovskite LSMCO and graphene nanoplates (L25G70, L50G45, and L75G20) were prepared and electrochemically characterized. The average initial specific capacitance values of L25G70, L50G45, and L75G20 were 50.11, 34.28, and 14.90 F g^-1^ at a scan rate of 5 mV s^-1^, and the standard deviation was 2.60, 2.41, and 1.95, respectively. During the cycling test at a scan rate of 20 mV s^-1^, the specific capacitance of each electrode maintained the specific capacitance with a small range of variation. However, L75G20 no longer exhibited electrochemical activity after 3000 cycles. The initial performance and cycling stability were excellent for L25G70. In particular, the analysis of the characteristics of L25G70 indicates that an increased lattice strain results in a high oxygen adsorption per the oxygen lattice. A large amount of anion adsorptive sites provides an enlarged space for SO4^2-^, thus the lattice distortion brings enhanced supercapcitive performance. These results suggest that supercapacitors employing composite electrodes fabricated using LSMCO and GNs are superior to perovskite-only electrodes and exhibit significant potential for commercialization, considering the stable anion adsorption behavior. Therefore, the LSMCO and GN composite developed in this study is a promising material to improve the cycling stability in supercapacitors.

## Methods

### Preparation of La_0.8_Sr_0.2_Mn_0.5_Co_0.5_O_3-δ_

La_0.8_Sr_0.2_Mn_0.5_Co_0.5_O_3-δ_ (LSMCO) as electrode material was synthesized via modified Pechini method^37^. The nitrate metal precursors of Lanthanum(III) nitrate hydrate (99.9%, Sigma Aldrich), strontium nitrate (98%, Samchun), manganese(II) nitrate tetrahydrate (97%, Sigma Aldrich), and cobalt(II) nitrate hexahydrate (97%, Samchun) as raw material were dissolved in 200 mL of deionized water at room temperature with stoichiometric ratio. The citric acid (CA, 99.5%, Samchun) and ethylenediaminetetraacetic acid (EDTA, 99.5%, Sigma Aldrich) as a chelating agent were added in nitrate solution with a molar ratio of which the metals, CA and EDTA is 1: 2.2: 2.2, respectively. The solution had a pH of 7 that was adjusted by ammonia solution (25%, Samchun) and was dehydrated at 573 K to form a vigorous gel and ignited to flame, resulting in the ash. The resulting ash-like material was pre-calcined at 673 K for 2 h to eliminate the organic component. Subsequently, annealing at 1273 K for 5 h was carried out to obtain pure and stoichiometric LSMCO powder.

### Fabrication of electrodes

The electrodes were prepared on a carbon paper cut to a dimension of 40 mm × 10 mm. According to the weight ratio of each electrode (Table 2), LSMCO, GNs (specific surface area 550 m^2^ g^-1^, Alfa Aesar), and polyvinylidene fluoride (PVDF, MTI Corporation, 2 mg) at a total mass of 40 mg were mixed in 0.2 ml dimethyl sulfoxide (DMSO, Sigma-Aldrich) by hand grinding for 30 min. 1.0–2.0 mg of the mixed active material was applied to the carbon paper in an area of 1 cm^2^, and heated for 15 min on a hot plate at 150 °C.

**Table 2:** Material ratios used in electrode fabrication.

Electrode	LSMCO (wt%)	Graphene (wt%)
L25G70	25	70
L50G45	50	45
L75G20	75	20

### Materials characterization

Morphology of the electrode was examined using field-emission scanning electron microscopy (FESEM; JEOL JXA-7600F), and the chemical composition was examined using EDS (X-Max, Oxford Instruments). XRD patterns of electrode materials were recorded using a D8 Advance (TRIO/TWIN) X-ray diffractometer. The specific surface area, total pore volume, and pore size distribution were measured using the BET method (BELSORPmini II). Surface chemical analysis was performed using XPS (Thermo ESCALAB 250) with Al Kα radiation. To confirm the elemental composition in the electrolyte after the cycling test, ICP-OES analysis was conducted using an Agilent Technologies 720 instrument.

### Electrochemical characterization

To evaluate the supercapacitor characteristics of the prepared electrodes based on GNs and LSMCO, CV, GCD, and EIS were performed in a three-electrode cell configuration using a potentiostat (VSP, Bio-Logic). The fabricated electrodes were used as working electrodes. A platinum wire, Hg/Hg_2_Cl_2_ electrode (saturated KCl), and 1 M H_2_SO_4_ (after purging with N_2_ gas for 20 min) were used as the counter electrode, reference electrode, and electrolyte, respectively. The CV and GCD experiments were performed in a voltage window between 0 and 0.8 V. CV was performed at scan rates of 5 and 20 mV s^-1^. GCD was measured at current densities from 0.5 to 10 A g^-1^. EIS was measured from 100 kHz to 100 mHz with an amplitude of 10 mV.

The specific capacitance (C_s_, F g^-1^) was calculated from the CV and GCD curves using Eqs. (1) and (2), respectively^38,39^.

1 $$C_{s} = \frac{1}{s \cdot m \cdot \Delta V}\mathop \smallint \limits_{{V_{1} }}^{{V_{2} }} idV$$

2 $$C_{s} = I \cdot t_{d} /\left( {m \cdot \Delta V} \right)$$

where *s* is the scan rate; *m* is the mass of the active material; *i* is the response current; *ΔV * is the voltage window; *V*_*1*_ is the lower voltage limit; *V*_*2*_ is the upper voltage limit; *I* is the discharge current; *t*_*d*_ is the discharge time.

The gravimetric power (*P*) and energy density (*E*) were calculated using Eqs. (3) and (4), respectively^40^.

3 $$E = CV^{2} /\left( {2 \times 3.6} \right)$$

4 $$P = E/t \times 3600$$

where *C* is the specific capacitance of the symmetric system; *V* is the voltage change during the discharge process in the *V–t* curve; *t*_*d*_ is the discharge time.

## Supplementary Information

Supplementary Information.
