# Supplementary material for: Structurally distorted perovskite La0.8Sr0.2Mn0.5Co0.5O3-δ by graphene nanoplatelet and their composite for supercapacitors with enhanced stability
Source: Sci Rep. 2022 Jun 16;12:10043. doi: 10.1038/s41598-022-14324-5 (PMC9203535; doi:10.1038/s41598-022-14324-5)
Supplement: Supplementary file 1 — Supplementary Information. [file 41598_2022_14324_MOESM1_ESM.docx]

**Supplementary Information**

**Structurally distorted perovskite La_0.8_Sr_0.2_Mn_0.5_Co_0.5_O_3-δ_ by graphene nanoplatelet and their composite for supercapacitors with enhanced stability**

Bo-Min Kim ^1,*^, Hyo-Young Kim ^2,*^, Sung-Wan Hong ^3^, Won Ho Choi ^4+^, Young-Wan Ju ^2,5,+^, and Jeeyoung Shin^1,4,+^

*^1^ Department of Mechanical Systems Engineering, Sookmyung Women’s University, Seoul 04310, Korea*

*^2^ Department of Chemical Engineering, College of Engineering, Wonkwang University, Iksan, Jeonbuk 54538, Korea*

*^3^ Department of Electronics Engineering, Sogang University, Seoul 04107, Korea*

*^4^ Institute of Advanced Materials and Systems, Sookmyung Women’s University, Seoul 04310, Korea*

*^5^ Nanoscale Sciences and Technology Institute, Wonkwang University, Iksan, Jeonbuk 54538, Korea*

*^+^Corresponding authors: wonhochoi@sookmyung.ac.kr (W.H.C.); ywju1978@wku.ac.kr (Y.-W.J); jshin@sookmyung.ac.kr (J.S.).***^*^** *These authors contributed equally to this work*

**Figure S1.** Energy-dispersive X-ray spectroscopy (EDS) elemental mapping images of L25G70, L50G45, and L75G20.

**Figure S2.** SEM and EDS images of L25G70, L50G45 and L75G20 after cycling test

**Figure S3.** CV curve at a scan rate of 5 mV s^-1^

**Figure S4.** CV curve of GN at a scan rate of 20 mV s^-1^ during 5000 cycles

**Figure S5.** CV curve of L25G70 at a scan rate of 20 mV s^-1^ during 5000 cycles

**Figure S6.** CV curve of L50G45 at a scan rate of 20 mV s^-1^ during 5000 cycles

**Figure S7.** CV curve of L75G20 at a scan rate of 20 mV s^-1^ during 3000 cycles

**Figure S8.** Galvanostatic charge-discharge (GCD) curves of GN ^1^

**Figure S9.** Galvanostatic charge-discharge (GCD) curves of L25G70

**Figure S10.** Galvanostatic charge-discharge (GCD) curves of L50G45

**Figure S11.** Galvanostatic charge-discharge (GCD) curves of L75G20

**Figure S12**. Comparison of electrochemical performance of pseudo-capacitive materials

**Figure S13.** Nyquist plot of GN


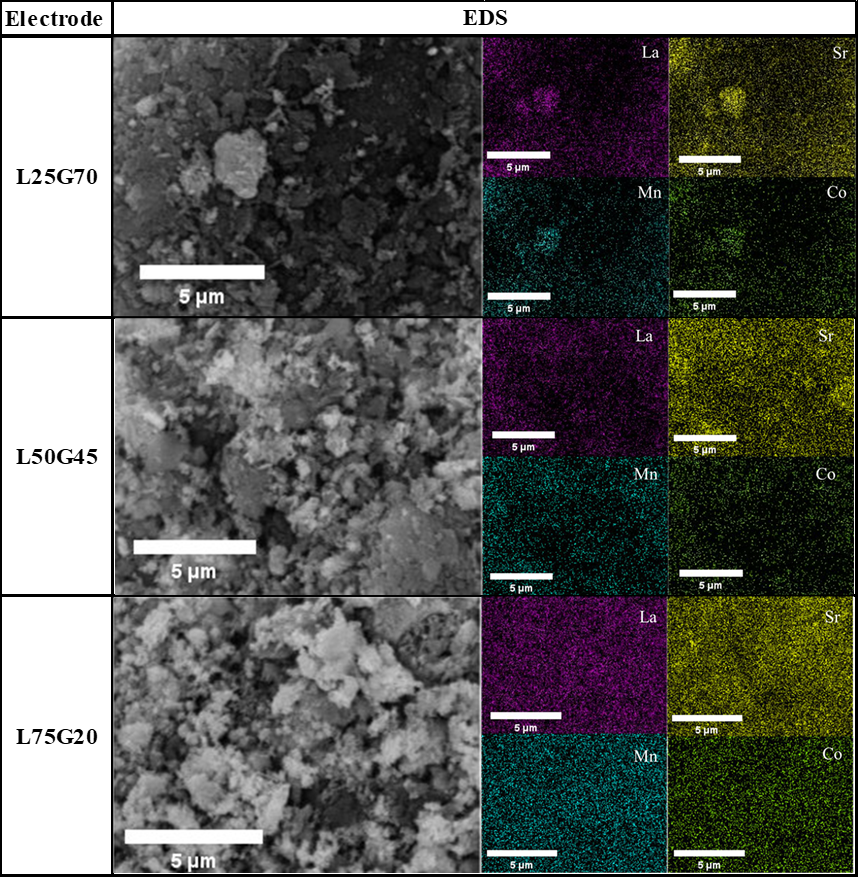


**Figure S1.** Energy-dispersive X-ray spectroscopy (EDS) elemental mapping images of L25G70, L50G45, and L75G20.

**Figure S2.** SEM and EDS images of L25G70, L50G45 and L75G20 after cycling test


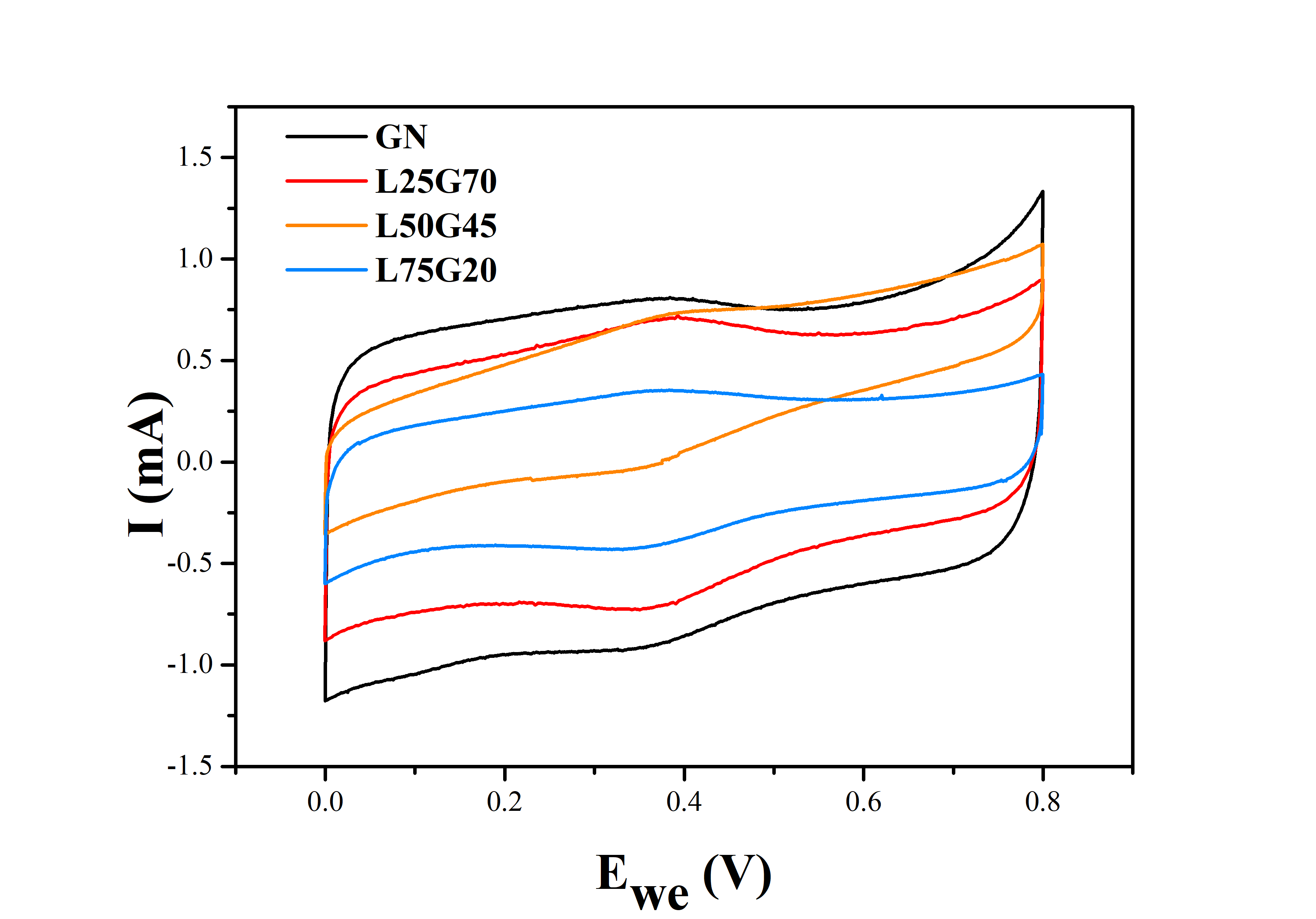


**Figure S3.** CV curve at a scan rate of 5 mV s^-1^


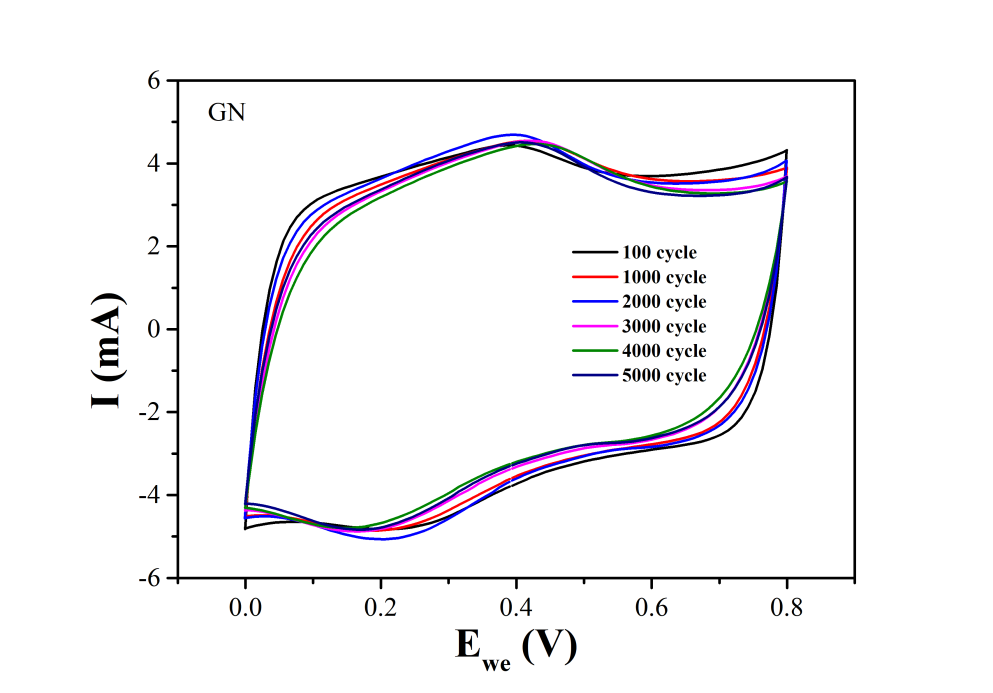


**Figure S4.** CV curve of GN at a scan rate of 20 mV s^-1^ during 5000 cycles


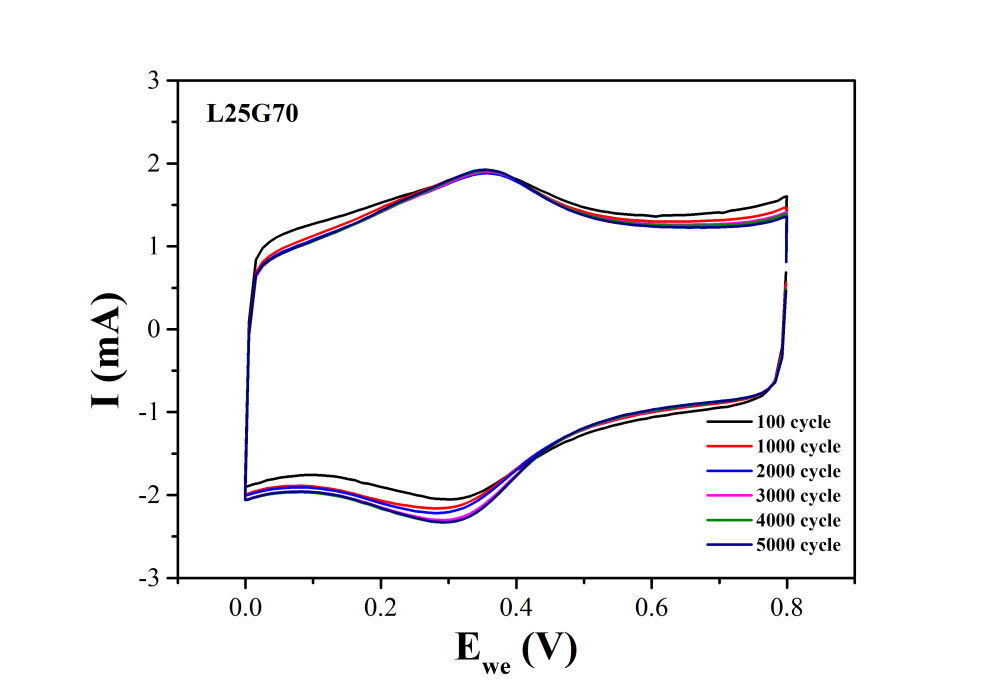


**Figure S5.** CV curve of L25G70 at a scan rate of 20 mV s^-1^ during 5000 cycles


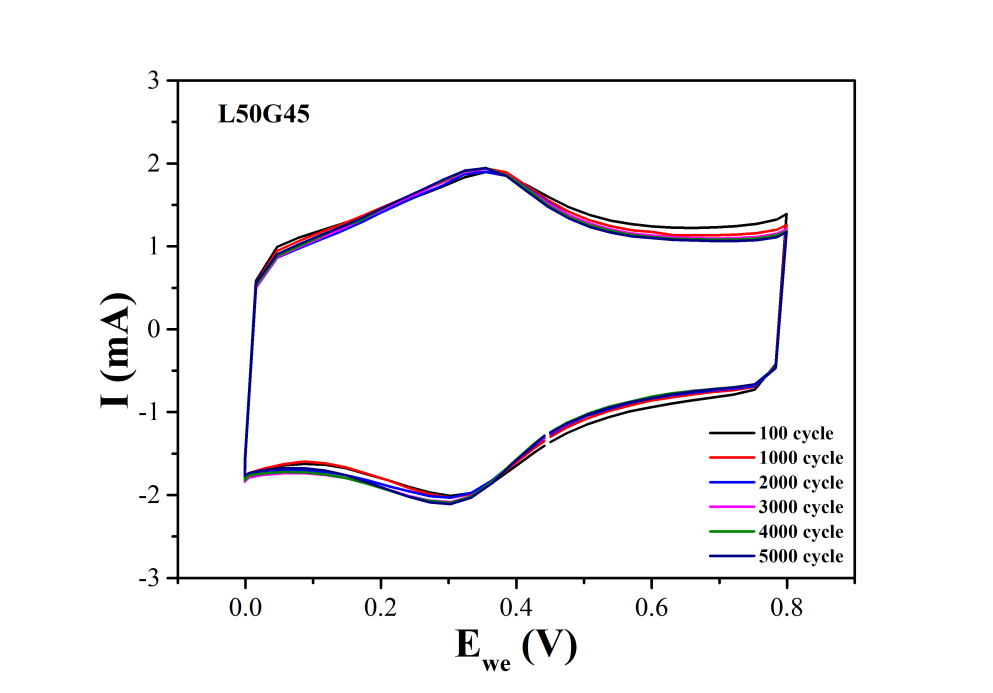


**Figure S6.** CV curve of L50G45 at a scan rate of 20 mV s^-1^ during 5000 cycles


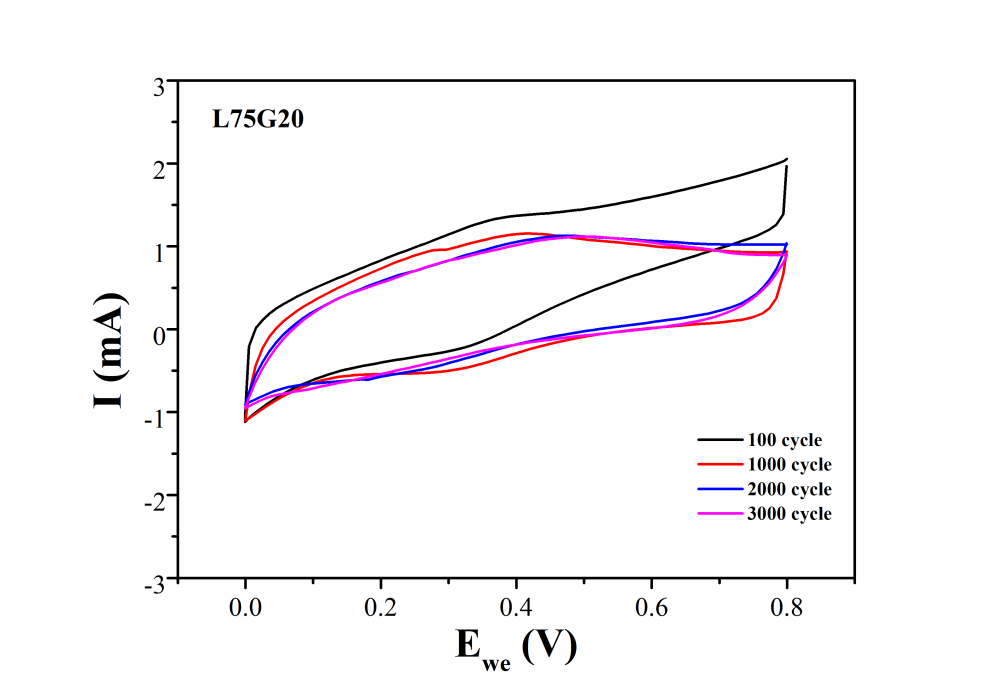


**Figure S7.** CV curve of L75G20 at a scan rate of 20 mV s^-1^ during 3000 cycles


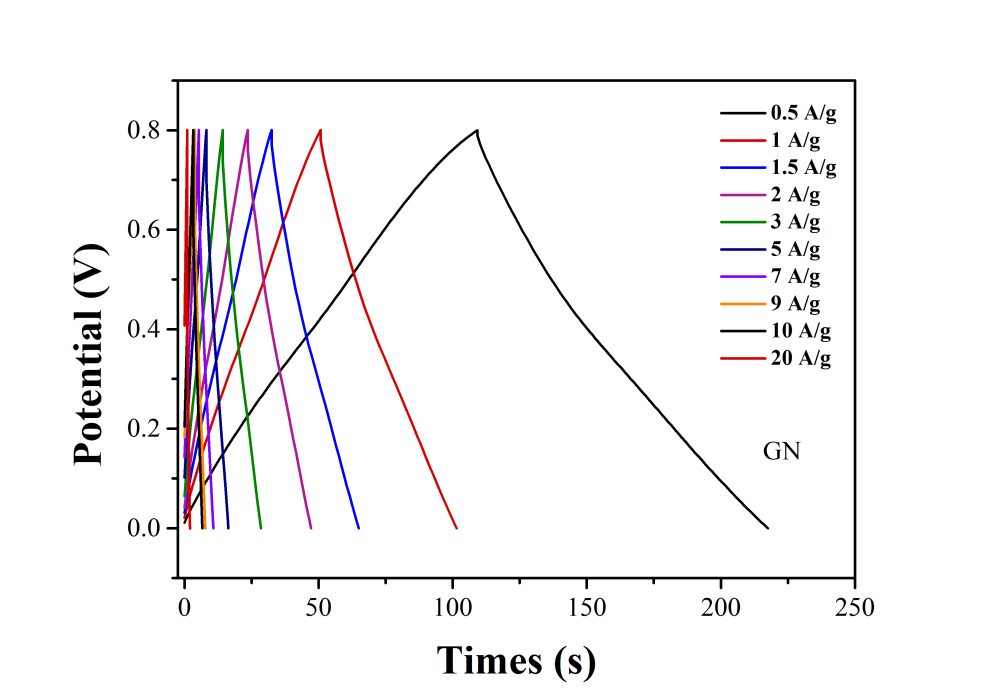


**Figure S8.** Galvanostatic charge-discharge (GCD) curves of GN


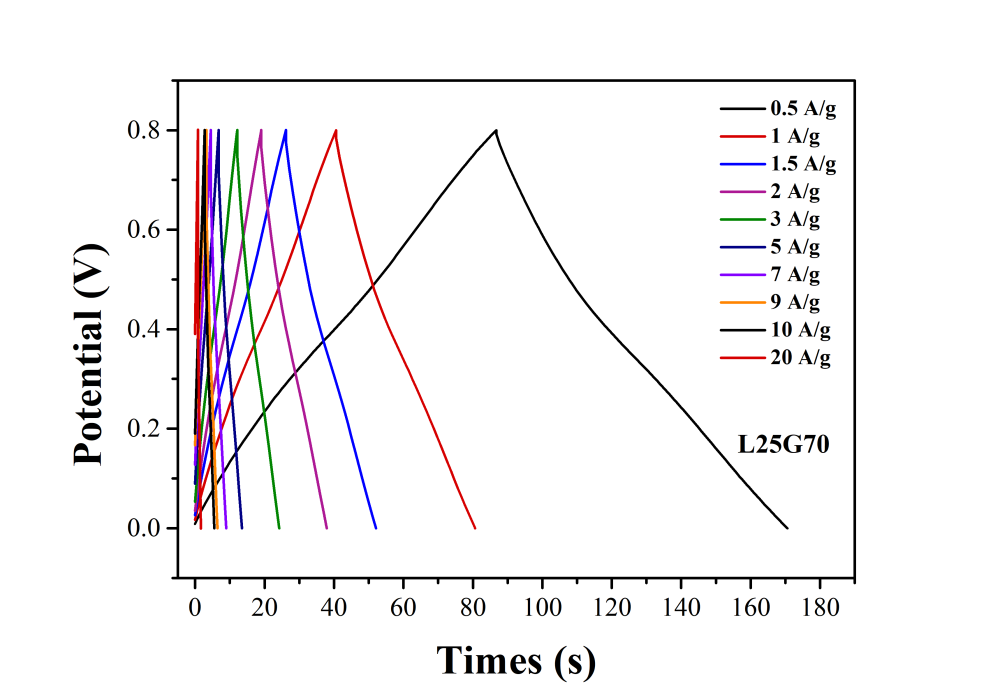


**Figure S9.** Galvanostatic charge-discharge (GCD) curves of L25G70


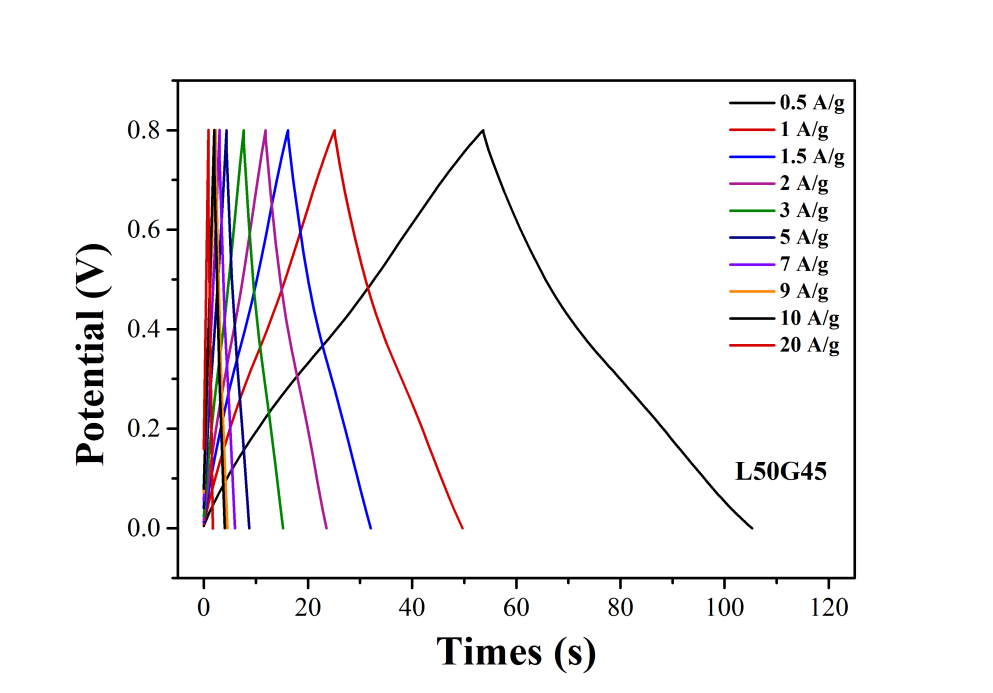


**Figure S10.** Galvanostatic charge-discharge (GCD) curves of L50G45


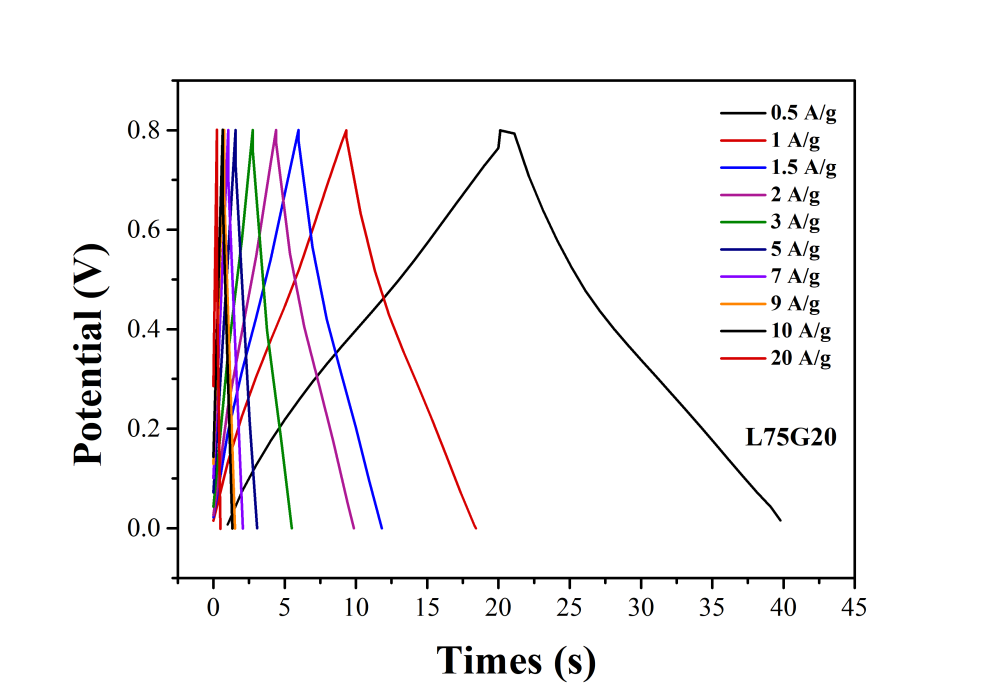


**Figure S11.** Galvanostatic charge-discharge (GCD) curves of L75G20

**Figure S12**. Comparison of electrochemical performance of pseudo-capacitive materials ^1,2,3^


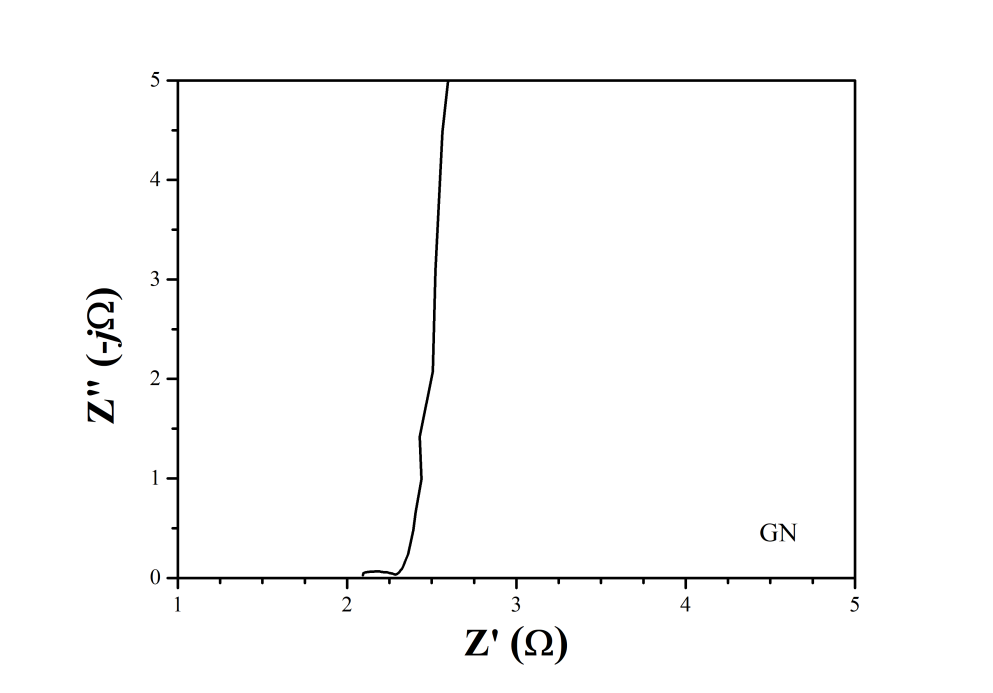


**Figure S13.** Nyquist plot of GN

**References**

1. MASALOVICH, M., et al. Development of pseudocapacitive materials based on cobalt and iron oxide compounds for an asymmetric energy storage device. *Electrochimica Acta*, **410**, 139999 (2022).

2. ZHANG, Xu, et al. Nanosheet-assembled NiCo-LDH hollow spheres as high-performance electrodes for supercapacitors. J. Colloid Interface Sci, **606**, 1120-1127 (2022)

3. WEI, Yudi, et al. All pseudocapacitive MXene-MnO2 flexible asymmetric supercapacitor. *J. Energy Storage*, **45**, 103715 (2022)
